# Optogenetic activation of serotonergic terminals facilitates GABAergic inhibitory input to orexin/hypocretin neurons

**DOI:** 10.1038/srep36039

**Published:** 2016-11-08

**Authors:** Srikanta Chowdhury, Akihiro Yamanaka

**Affiliations:** 1 Department of Neuroscience II, Research Institute of Environmental Medicine, Nagoya University, Nagoya 464-8601, Japan

## Abstract

Orexin/hypocretin neurons play a crucial role in the regulation of sleep/wakefulness, primarily in the maintenance of wakefulness. These neurons innervate wide areas of the brain and receive diverse synaptic inputs including those from serotonergic (5-HT) neurons in the raphe nucleus. Previously we showed that pharmacological application of 5-HT directly inhibited orexin neurons via 5-HT1A receptors. However, it was still unclear how 5-HT neurons regulated orexin neurons since 5-HT neurons contain not only 5-HT but also other neurotransmitters. To reveal this, we generated new triple transgenic mice in which orexin neurons express enhanced green fluorescent protein (EGFP) and 5-HT neurons express channelrhodopsin2 (ChR2). Immunohistochemical studies show that nerve endings of ChR2-expressing 5-HT neurons are in close apposition to EGFP-expressing orexin neurons in the lateral hypothalamic area. Using these mice, we could optogenetically activate 5-HT nerve terminals and record postsynaptic effects from orexin neurons. Activation of nerve terminals of 5-HT neurons directly inhibited orexin neurons via the 5HT1A receptor, and also indirectly inhibited orexin neurons by facilitating GABAergic inhibitory inputs without affecting glutamatergic inputs. Increased GABAergic inhibitory inputs in orexin neurons were confirmed by the pharmacological application of 5-HT. These results suggest that orexin neurons are inhibited by 5-HT neurons, primarily via 5-HT, in both direct and indirect manners.

Orexin (also known as hypocretin)-producing neurons (orexin neurons) are exclusively distributed in the perifornical area and the lateral hypothalamic area (LHA)^1^,^2^,^3^. Orexin functions as a neuropeptide that binds to G-protein coupled receptors (GPCRs), termed orexin 1 (OX1R) and orexin 2 (OX2R) receptors, and participates in multiple physiological responses including feeding behaviour, sleep/wakefulness, and perception of pain^1^,^4^,^5^,^6^. To regulate such intricate physiological responses, orexin neurons receive and integrate diverse synaptic inputs from many brain regions, including serotonergic neurons in the midbrain raphe nuclei^7^.

Ascending serotonin (5-hydroxytryptamine, 5-HT) neurons are mostly concentrated in two important nuclei, the dorsal raphe (DR) and the median raphe (MnR)^8^ nuclei in the mammalian central nervous system (CNS). These areas have long been known to be involved in multiple physiological and behavioural conditions including cognition, motor activity, pain modulation, food intake, energy balance, and circadian rhythm^9^,^10^,^11^,^12^,^13^. However, the function of 5-HT on the sleep/wakefulness cycle is complex and controversial. Although 5-HT was first hypothesized to induce drowsiness and sleep, it is now well-established to be wake promoting and to inhibit rapid eye movement (REM) sleep^14^. Retrograde labelling to reveal input pathways to 5-HT neurons showed that 5-HT neurons in the DR receive direct synaptic inputs from orexin neurons^15^. Moreover, an *in situ* hybridization study revealed that the raphe nucleus expresses both OX1R and OX2R^16^. Orexin neurons modulate the activity of 5-HT neurons in the raphe in both a direct and indirect manner. The direct modulation induces an excitatory effect via both OX1R and OX2R and the indirect modulation induces an inhibitory effect that is mediated through increasing inhibitory input from GABAergic interneurons^17^. In contrast, anatomical studies have revealed descending neural pathways to orexin neurons including from 5-HT neurons^7^. A patch clamp study using brain slices from orexin-enhanced green fluorescent protein (*orexin-EGFP*) mice revealed that pharmacologically applied 5-HT directly hyperpolarized orexin neurons by increasing potassium conductance, and this response was inhibited by a specific 5-HT1A receptor antagonist^18^. This study suggested involvement of 5HT1A receptors and subsequent activation of G protein-coupled inwardly-rectifying potassium (GIRK) channels in orexin neurons. Orexin neurons receive diversified inputs from local interneurons, and the activity of orexin neurons is controlled by both negative and positive feedback neural networks^19^. However, how these local glutamatergic and GABAergic interneurons that directly innervate orexin neurons are regulated by 5-HT neurons remains unknown. Additionally, it is reported that 5-HT neurons not only contain 5-HT but also release other co-neurotransmitters, such as acetylcholine, catecholamine, glutamate, aspartate, GABA, and also the neuropeptide substance P^20^,^21^,^22^,^23^. It is also well established that 5-HT can function as a neuromodulator of glutamate and GABA, which are principal molecules that mediate excitatory and inhibitory signals in the mammalian CNS, respectively^24^. Thus, the comprehensive effect of 5-HTergic regulation of orexin neurons could be complex and is largely unknown.

To help reveal these mechanisms, we took advantage of optogenetics in this study. Optogenetics has become a very powerful technique for controlling neuronal membrane potential and firing^25^, and it has been used to target a specific projection or specific cell type to improve our current understanding of neuronal circuitry^26^. Using optogenetics, combined with *in vitro* slice patch clamp recording, we could specifically activate 5-HT nerve terminals in the LHA and record postsynaptic effects from orexin neurons. This study revealed that activation of 5-HT terminals in the LHA directly and indirectly inhibited the activity of orexin neurons.

## Results

### Triple transgenic (*Tg*) mice express EGFP in orexin neurons and ChR2 in 5-HT neurons

**Figure 1:**
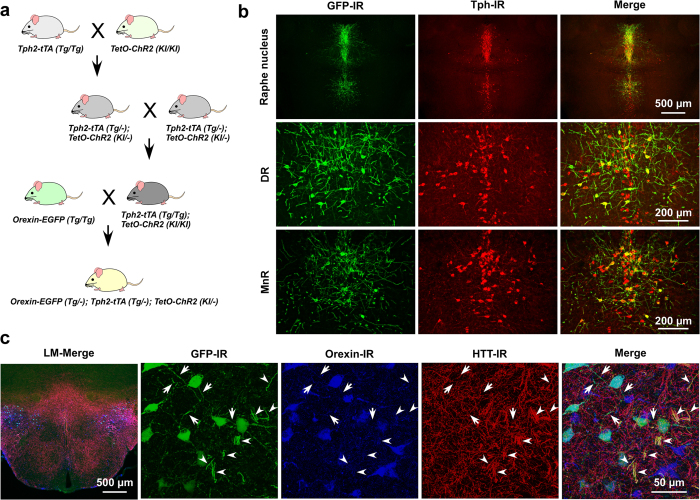
Triplegenic mice express EGFP exclusively in orexin and ChR2 exclusively in 5-HT neurons. **(a)** Breeding scheme to generate triplegenic mice. **(b)** Coronal brain slices containing raphe nucleus show co-localization of ChR2 (green) with 5-HT (red) in both DR and MnR. (**c)** Representative coronal sections of LHA from triplegenic mice. Sections were labelled with antibody for GFP (green), orexin (blue), and serotonin transporter, HTT (red). LM, lower magnification. Arrowheads indicate 5-HT nerve endings and arrows indicate orexinergic nerve terminals. These were in close apposition in the LHA.

To manipulate the nerve terminals of 5-HT neurons using optogenetics and to record the postsynaptic effects from orexin neurons, we generated a new line of *Tg* mice. We generated triple transgenic *orexin-EGFP; Tph2-tTA; TetO ChR2* mice (hereafter called triplegenic mice) (Fig. 1a). These triplegenic mice expressed EGFP in orexin neurons under control of the human prepro-orexin promoter^27^, and also a tetracycline-controlled transactivator (tTA) exclusively in 5-HT neurons in the raphe nucleus under the control of the Tph2 promoter. tTA binds to the tetracycline operator (TetO) sequence and induces ChR2 expression^28^. ChR2 was expressed as a fusion protein with enhanced yellow fluorescent protein (EYFP) to visualize ChR2-expressing neurons. We first confirmed the expression of ChR2 in 5-HT neurons and the expression of EGFP in orexin neurons via immunohistochemical studies. We stained brain slices containing the raphe nucleus with 2 different antibodies: anti-GFP antibody (that labels ChR2-expressing neurons) and anti-Tph antibody (that labels 5-HT-containing neurons). We also stained brain slices containing the LHA with 3 different antibodies: anti-GFP antibody (that labels both orexin neurons and ChR2-EYFP-expressing 5-HT nerve endings), anti-orexin A antibody (that labels orexin neurons in the LHA), and anti-serotonin transporter, 5-HT transporter (HTT) antibody (that labels cell bodies and nerve fibres of 5-HT neurons). In the triplegenic mice, ChR2 expression was observed in the raphe nucleus (MnR and DR) in the midbrain (Fig. 1b). We counted the number of 5-HT cell bodies that expressed ChR2. In the DR, 60.5 ± 2.4% of the total 2,385 counted cells, and in the MnR, 56.0 ± 3.8% of the total 1,223 counted 5-HT-positive neurons expressed ChR2-EYFP (*n* = 3). We found very little ectopic expression of ChR2 outside of the HTT-positive neurons. In the DR, 0.14 ± 0.12% of the cells, and in the MnR, 0.04 ± 0.04% of the 5-HT-negative neurons expressed ChR2-EYFP (*n* = 3). Comparatively, EGFP was exclusively expressed in orexin neurons (Fig. 1c). There was no ectopic expression of EGFP-immunoreactive neurons other than orexin-immunoreactive neurons. Confocal imaging revealed a dense projection of 5-HT nerve endings in the LHA. Moreover, cell bodies of orexin neurons and 5-HT nerve endings were in close apposition (arrowheads in Fig. 1c), supporting the hypothesis that 5-HT neurons regulate orexin neurons (Fig. 1c).

### Local application of 5-HT hyperpolarizes orexin neurons in brain slices of triplegenic mice

Next, we examined the basic electrophysiological properties of orexin neurons in newly generated triplegenic mice. By using *in vitro* slice patch clamp recording, we found that the firing frequency of orexin neurons in triplegenic mice was 2.4 ± 0.3 Hz (*n* = 21) and the average membrane capacitance of orexin neurons was recorded to be 30.7 ± 1.8 pF (*n* = 30) (Supplementary Figure 1a). The average resting membrane potential of orexin neurons was −54.4 ± 0.9 mV (*n* = 23). The average action potential amplitude, width at half-maximal amplitude, and area of spike of orexin neurons were found to be 84.3 ± 1.5 mV, 1.1 ± 0.04 ms, and 114.0 ± 3.8 mVms, respectively (*n* = 15) (Supplementary Figure 1b). These values were in good agreement with our previous recordings from *orexin-EGFP* mice^29^,^30^,^31^, suggesting that membrane properties of orexin neurons are conserved even in the triplegenic mice. We next confirmed the pharmacological effect of 5-HT on orexin neurons in the triplegenic mice. We recorded from orexin neurons in a whole-cell current-clamp configuration and applied 100 μM of 5-HT locally onto the brain slices. We found a sharp hyperpolarizing effect (−16.7 ± 1.3 mV) of 5-HT on orexin neurons that could completely silence the spontaneous action potentials of orexin neurons (*n* = 19) (Supplementary Figure 1c,d). All orexin neurons recorded were hyperpolarized by local application of 5-HT (100 μM). These data confirmed our previous result that the pharmacological effect of 5-HT is to hyperpolarize orexin neurons directly^18^.

### Blue light controls the activity of 5-HT neurons

A ChR2 variant (T159C with E123T, also called ChR2 (ET/TC)^32^) was used to control 5-HT neurons. ChR2 (ET/TC) has been previously reported to have very fast kinetics (τ_off_ = 8.1 ± 0.1 ms at −75 mV_hold_) and to induce larger photocurrents^33^. Thus, this ChR2 variant allows for single action potential generation even at high frequencies of blue light pulses (~40 Hz). We first sought to understand the illuminating conditions of blue light that would control 5-HT neurons expressing ChR2. 5-HT neurons were whole cell patch-clamped and voltage-clamped at −60 mV. Blue light (475 ± 17.5 nm) illumination induced an inward photocurrent that depended on light intensity (Supplementary Figure 2a,b). For example, 100% (15.4 mW) of blue light induced −545.6 ± 40.5 pA of transient inward current and −246.1 ± 16.3 pA of sustained inward current (*n* = 11). 5-HT neurons also exhibited a light intensity-dependent increase in firing frequencies upon continuous illumination of blue light for 1 sec (Supplementary Figure 2c,e). 100% of blue light intensity increased the firing frequency to 256.4 ± 27.6%, whereas 50% intensity increased it to 251.8 ± 22.3% (*n* = 11). Although 5-HT neurons from triplegenic mice showed single faithful spikes upon illuminating high-frequency spike trains of brief light pulses (1 ms width) (Supplementary Figure 2d,f), increasing frequency gradually decreased firing probability. For example, blue light pulse trains of 10% intensity and 40 Hz frequency reduced the firing probability to 57.4 ± 6.6% (*n* = 10). Brain slices including the LHA contain 5-HT nerve terminals but not 5-HT neuron cell bodies. Thus, these 5-HT nerve endings in brain slices including the LHA are deprived of spontaneous firings. Therefore, we decided to activate 5-HT nerve endings in the LHA via continuous illumination of blue light instead of high-frequency blue light pulses for subsequent experiments.

### Photo-stimulation of 5-HT nerve endings facilitates GABAergic, but not glutamatergic, inputs to orexin neurons

**Figure 2:**
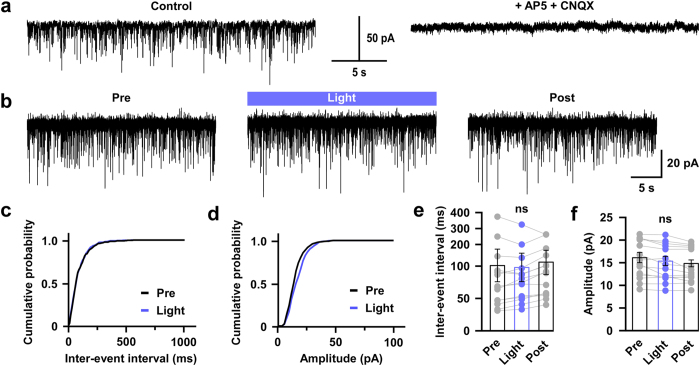
Activation of serotonergic nerve endings did not affect glutamatergic inputs onto orexin neurons. **(a)** EPSCs were recorded from orexin neurons at −60 mV_hold_ in the presence of intracellular QX-314 (1 mM) and extracellular picrotoxin (400 μM). EPSCs were completely blocked by adding NMDA and AMPA receptor antagonist, namely AP-5 (50 μM) and CNQX (20 μM), respectively. **(b)** Traces showing the effect of continuous blue light illumination of 50% intensity for 30 sec. Representative cumulative probability plot shows that activation of 5-HT nerve endings did not change EPSC frequency **(c)** and amplitude **(d)**. Combined bar and scatter plots summarize the data for EPSC **(e)** interval (*p* = 0.93 vs pre, 0.79 vs post) and **(f)** amplitude (*p* = 0.62 vs pre, 0.73 vs post) (*n* = 14). Data are provided as mean ± s.e.m. (*ns*, not significant; *p* value was calculated by one-way ANOVA followed by post hoc Fisher’s least significant difference (LSD) test).

**Figure 3:**
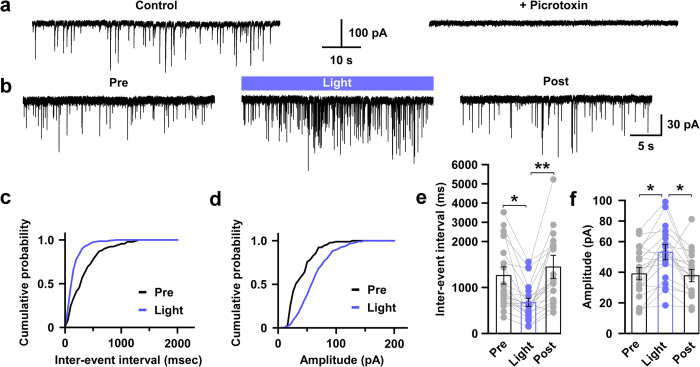
Activation of serotonergic nerve endings increase GABAergic inputs to orexin neurons. **(a)** IPSCs were recorded from orexin neurons at −60 mV_hold_ in the presence of intracellular QX-314 (1 mM) and extracellular AP-5 (50 μM) and CNQX (20 μM). IPSCs were blocked by adding picrotoxin (400 μM), a GABA-activated chloride channel blocker. (**b**) Traces showing the effect of continuous blue light illumination of 50% intensity for 30 sec on brain slices. Representative cumulative probability plot for IPSC frequency **(c)** and amplitude **(d)** shows decreased input interval and increased input amplitude. Combined bar and scatter plots summarize the data for IPSC interval **(e)** (*p* values are 0.03 vs pre and 0.005 vs post) and amplitude **(f)** (*p* = 0.02 vs pre and 0.01 vs post) (*n* = 20). Data are provided as mean ± s.e.m. *p < 0.05, **p < 0.01. *p* values were calculated by one-way ANOVA followed by post hoc fisher’s LSD test.

To evaluate the effect of 5-HT nerve ending activation on orexin neurons, we prepared acute coronal brain slices containing the hypothalamus from triplegenic mice and recorded spontaneous EPSCs and IPSCs from postsynaptic orexin neurons at a holding potential of −60 mV. EPSCs were recorded in the presence of 400 μM of extracellular picrotoxin (PTX), a GABA_A_ receptor antagonist, and IPSCs were recorded in the presence of extracellular AP-5 (50 μM) and CNQX (20 μM), glutamate receptor antagonists. EPSCs and IPSCs were confirmed by adding AP-5 and CNQX (Fig. 2a) or picrotoxin (Fig. 3a) in the perfused extracellular solution, respectively. Pipette solutions contained 1 mM of QX-314 to inhibit voltage gated sodium channels to block action potential generation. In this recording condition, both EPSCs and IPSCs were recorded as inward current. Optogenetic activation of 5-HT terminals in brain slices did not affect EPSC input to orexin neurons (Fig. 2a–f). Neither the inter-event interval nor the amplitude of EPSC input changed significantly upon activating 5-HT terminals. The average EPSC interval at baseline (*pre*) was 100.9 ± 27 ms, whereas that after turning on blue light (*light*) was 97.9 ± 21.9 ms; *n* = 14, *p* = 0.93. Again, the average *pre* EPSC amplitude was 16.1 ± 1.1 pA whereas that during *light* was 15.4 ± 1.1 pA; *n* = 14, *p* = 0.62 (Fig. 2c–f). We recorded EPSCs from 21 orexin neurons obtained from 3 different mice and randomly analysed 14 of them. In contrast to excitatory system findings, when we recorded inhibitory GABAergic inputs to orexin neurons and activated 5-HT nerve endings by illuminating blue light, we found a dramatic increase in IPSCs in orexin neurons (Fig. 3b). The inter-event intervals of IPSC inputs were decreased (*pre* was 1264.4 ± 191.8 ms and *light* was 684.1 ± 93.6 ms; *n* = 20, *p* < 0.05), implying an increase in total inhibitory input (Fig. 3e), while the amplitudes of IPSC inputs were also increased (*pre* was 39.3 ± 3.8 pA and *light* was 52.1 ± 4.6 pA; *n* = 20, *p* < 0.05) (Fig. 3f). However, IPSCs generated by brief blue light pulses were not generated in a precise time-locked manner and continued even after the cessation of light (Supplementary Figure 3). We also measured the delay in the increased IPSCs from the time of illumination-onset and found it to be 7.32 ± 1.65 s (*n* = 18). This phenomenon supports the idea that 5-HT neurons modulate the function of their targets primarily by passive diffusion in a volume transmission manner in many brain regions^24^,^34^. Again, 20 out of 45 neurons recorded from 7 different mice (≥1 neuron/mouse) showed increased IPSCs; this variability may arise from the fact that only a fraction of 5-HT neurons in the DR and MnR expressed ChR2 in these mice. Taken together, these data suggest that photo-stimulation of 5-HT nerve terminals influences post-synaptic current of orexin neurons by facilitating GABAergic transmission, without affecting EPSCs.

### 5-HT neurons produce a direct inhibitory effect on orexin neurons via the 5-HT1A receptor

**Figure 4:**
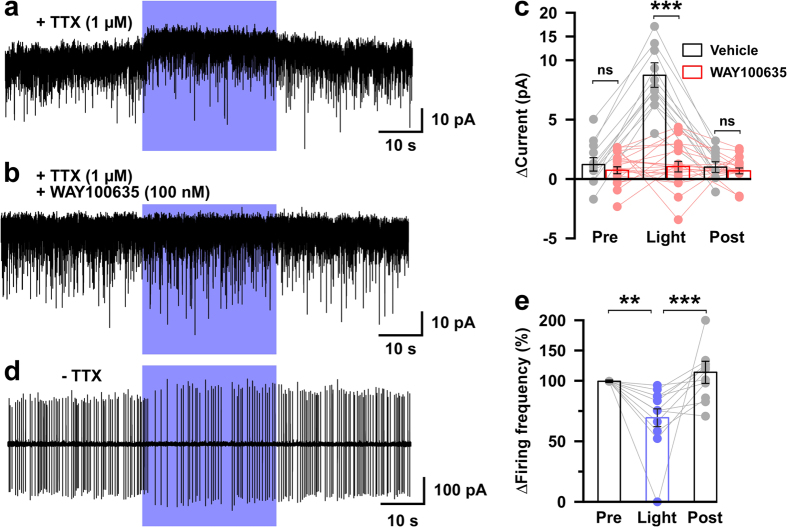
Activation of 5-HT nerve endings induced an outward current and decreased the firing rate of orexin neurons in triplegenic mice. **(a–c)** Outward currents recorded from orexin neurons at −60 mV_hold_ in the presence of 1 μM extracellular TTX were abolished in the presence of the 5-HT1A antagonist, WAY100635. Representative trace showing the light evoked outward current **(a)**, which was blocked by WAY100635 **(b)**. **(c)** Combined bar and scatter plot summarizing the data in **a** and **b**; vehicle, *n* = 12; WAY100635, *n* = 21; vehicle vs WAY100653, *p* = 3.1 × 10^−19^ (light), 0.46 (pre), 0.58 (post). **(d,e) S**pontaneous firing of orexin neurons was recorded by loose cell-attached recording. **(d)** Representative trace showing the decreased firing induced by light. **(e)** Combined bar and scatter plot summarizing data in **d** (*n* = 14; *p* = 0.004 vs pre; and 5 × 10^−4^ vs post). **p < 0.01, ***p < 0.001. *p* values were calculated by one-way ANOVA followed by a post hoc Fisher’s LSD test.

We next tested the complete effect of activation of 5-HT nerve endings on the activity of orexin neurons. We recorded postsynaptic currents from orexin neurons in brain slices of triplegenic mice at a holding potential of −60 mV in the presence of extracellular tetrodotoxin (TTX, 1 μM), which blocks voltage-gated sodium channels and the generation of action potentials, thus eliminating spontaneous events generated by neuronal firings. We found a small but robust effect on orexin neurons by 5-HT terminal activation (Fig. 4a,c). Blue light illumination (50% intensity) induced an outward current in orexin neurons (8.7 ± 1.1 pA; *n* = 12, *p* < 0.001 vs *pre*). This effect of 5-HT nerve terminal activation on orexin neurons was dependent on the light intensity (Supplementary Figure 4), and was observed in 12 out of 22 neurons recorded from 4 different mice (≥1 neuron/mouse). However, the inhibitory effect was reduced to 1.02 ± 0.4 pA when acute brain slices were treated with the 5-HT1A receptor selective antagonist, WAY100635 (*n* = 21, *p* < 0.001 vs vehicle; Fig. 4b–c). Brain slices were pretreated with 100 nM of WAY100635 for 2 min prior to illumination. We then performed loose cell-attached recordings from orexin neurons to reveal the effects on firing frequency in the absence of TTX. Blue light illumination (50% intensity) significantly decreased the firing frequency compared with before (*pre*) or after (*post*) light illumination (70.1 ± 7.2%; *n* = 14, *p* < 0.01 vs *pre*) (Fig. 4d,e). Taken together, these findings clearly indicate a direct inhibitory effect of 5-HT neurons on orexin neurons mediated by the 5-HT1A receptor.

### 5-HT application produced a similar effect on orexin neurons as 5-HT terminal activation

**Figure 5:**
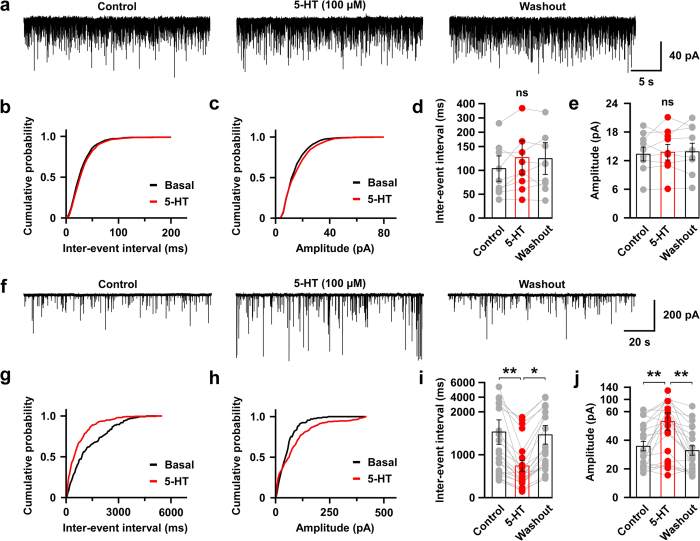
Local application of 100 μM of 5-HT mimics the effect of light evoked activation of 5-HT nerve endings. **(a–e)** EPSCs were recorded from orexin neurons at −60 mV_hold_ in the presence of intracellular QX-314 (1 mM) and extracellular picrotoxin (400 μM). (**a**) Traces showing the effect of local application of 100 μM of 5-HT on EPSCs. Representative cumulative probability plot shows that local application did not change EPSC frequency **(b)** and amplitude **(c)**. Bar and scatter plot summarizes the data on EPSC interval **(d)** (*p* = 0.60 vs pre, 0.97 vs post) and amplitude **(e)** (*p* = 0.86 vs pre, 0.92 vs post) (*n* = 9). **(f–j)** IPSCs were recorded from orexin neurons at −60 mV_hold_ in the presence of intracellular QX-314 (1 mM) and extracellular AP-5 (50 μM) and CNQX (20 μM). **(f)** Traces showing the effect of local application of 100 μM of 5-HT on IPSCs. Representative cumulative probability plot shows the effect of local application on the inter-event interval **(g)** and amplitude **(h)**. Bar and scatter plot summarizes the data on the IPSC interval **(i)** (*p* = 0.007 vs pre, 0.02 vs post) and amplitude **(j)** (*p* = 0.007 vs pre, 0.002 vs post) (*n* = 21). *ns*, not significant; *p < 0.05, **p < 0.01, ***p < 0.001. *p* values were calculated by one-way ANOVA followed by a post hoc Fisher’s LSD test).

Using acute coronal brain slices generated from triplegenic mice, we next recorded EPSCs and IPSCs from orexin neurons at −60 mV_hold_ and locally applied 5-HT (100 μM), dissolved in the bath solution. We found that 5-HT application significantly increased IPSC frequency and amplitude but did not affect EPSCs in orexin neurons (Fig. 5a–j). The average EPSC interval at baseline was 104.1 ± 25.6 ms whereas that during local application of 100 μM of 5-HT was 126.0 ± 34.1 ms; *n* = 9, *p* > 0.05 (Fig. 5d). Again, the average EPSC amplitude at baseline was recorded as 13.4 ± 1.4 pA whereas that observed during 5-HT application was 13.8 ± 1.6 pA; *n* = 9, *p* > 0.05 (Fig. 5e). In the case of IPSC recordings, the average interval at baseline was 1526.5 ± 276.3 ms whereas that during 5-HT application was 716.9 ± 12.5 ms; *n* = 22, *p* < 0.01 (Fig. 5i). Similarly, the average IPSC amplitude also increased significantly. At baseline it was 35.4 ± 3.3 pA whereas during 5-HT it was 52.0 ± 5.8 pA; *n* = 22, *p* < 0.01 (Fig. 5j). Thus, the regulation of orexin neurons by 5-HT showed a strong resemblance to that induced by photoactivation of 5-HT nerve terminals. These data indicate that 5-HT is the major neurotransmitter released by 5-HT neurons to regulate orexin neuron function in the LHA.

## Discussion

Our previous study showed that pharmacologically applied 5-HT directly hyperpolarized orexin neurons via 5-HT1A receptors and GIRK channels^18^. Here, we revealed (using optogenetic activation of 5-HT nerve endings in the hypothalamus) that 5-HT neurons directly inhibit orexin neurons and also indirectly inhibit them by increasing functional GABAergic input from interneurons in the LHA.

Although orexin had initially been identified as an important neuropeptide that regulates feeding behaviour^1^, the peptide was subsequently found to be crucial in maintaining wakefulness^35^. It was also found that ablation of orexin neurons can produce narcolepsy-like behaviour and disruption of sleep patterns^36^. These data suggest that the activity of orexin neurons depends immensely on sleep/wakefulness cycles, remaining active during wake states and silent during sleep^37^,^38^. To maintain such on/off conditions, orexin neurons feature a complex integration system between excitatory and inhibitory inputs from other brain areas^7^. A wide range of brain areas in the mammalian CNS has already been identified as upstream neuronal populations that innervate orexin neurons. These areas include the basal forebrain, amygdala, lateral septum, ventrolateral preoptic nucleus, periaqueductal gray matter, posterior/dorsomedial hypothalamus, ventral tegmental area, lateral parabrachial nucleus, and raphe nuclei^39^,^40^. Regarding the raphe nucleus, orexin neurons densely innervate and directly and indirectly modulate these neurons via both OX1R and OX2R. In addition, we show here that 5-HT neurons in the raphe nucleus densely innervate the hypothalamus and inhibit the activity of orexin neurons. Previously, we generated transgenic mice in which orexin neurons overexpress 5-HT1A receptors using the tet-off system^41^. Overexpression of 5-HT1A receptors in orexin neurons, which increased the efficiency of inhibitory inputs from 5-HT neurons, induced fragmentation of sleep/wakefulness in mice, which suggested that this neural circuit functions in a negative feedback manner. Our current findings further support this idea, whereby 5-HT neurons directly and indirectly inhibit orexin neurons to function as a negative feedback circuit.

Loss of orexin neurons is associated with the chronic sleep disorder narcolepsy^1^,^2^. Symptoms of narcolepsy are characterized by sleep fragmentation, excessive daytime sleepiness, hypnagogic hallucinations, and cataplexy. A recent study using mice that lack both orexin receptors (OX1R^−/−^ OX2R^−/−^) showed that specific restoration of OX2R in DR neurons entirely prevented cataplexy-like episodes, and this rescue was correlated with the number of 5-HT neurons restored with orexin receptors in the DR^42^. These findings suggest that regulation of 5-HT neuron activity by orexin neurons is enough to prevent cataplexy, and that this efferent pathway of the orexin neuron system could represent a major target to study the pathophysiology of narcolepsy. Although orexin neurons in the LHA have been reported to be innervated by numerous 5-HT terminals, the anatomical sources of this 5-HT input remain controversial. Sakurai *et al*. used a genetic tracing system (expressing the c-terminal fragment of tetanus toxin in orexin neurons) to identify upstream neuronal populations in mice, and showed that orexin innervation is mostly associated with 5-HT neurons residing in the median and paramedian raphe nuclei^39^. In comparison, Yoshida *et al*. injected cholera toxin B, a retrograde tracer, in the LHA of rat brain and showed that both the DR and MnR strongly innervated the LHA^40^. In the present study, we used *Tg* mice in which 5-HT neurons of both the DR and MnR expressed ChR2, and thus, we could not distinguish nerve endings from the DR or MnR. Expression of ChR2 via viral mediated gene delivery systems in either the DR or MnR could reveal which subareas of the raphe nuclei are involved in the inhibition of orexin neurons. Again, one potential caveat of this study is necessity of blue light illumination for searching EGFP expressing orexin neurons which was also used for optogenetic excitation. This is partly due to limited availability of transgenic mice expressing red fluorescent protein under control of prepro-orexin promoter. Alternatively, it could be addressed by expressing red-shifted channelrhodopsin (ChR2) such as VChR1, ChR1/VChR1, ReaChR etc. in 5-HT neurons. However, the fact that red-shifted ChR2 are also activated by blue light greatly minimizes its usefulness here^43^,^44^,^45^.

5-HT performs its physiological functions through its 14 receptor subtypes. Except for the 5-HT3 receptor, an ion channel, all other subtypes are GPCRs^24^. Therefore, it is no surprise that the regulatory mechanism underlying sleep/wakefulness by 5-HT neurons is complex. Activation of endogenous 5-HT nerve endings directly regulates orexin neuron function by activating the 5-HT1A receptor, which is a GPCR coupled with Gi/Go, mediating an inhibitory effect on orexin neurons. However, 5-HT1A is not the only 5-HT receptor involved in sleep/wakefulness regulation. Studies using receptor subtype-selective knock-out mice already reported that 5-HT1B, 5-HT2A, and 5-HT2C are also involved in this paradigm^46^,^47^,^48^. Moreover, it is likely that several 5-HT receptors are involved in modulating different stages of sleep. For example, 5-HT1A receptors were reported to enhance wakefulness and rapid eye movement (REM) sleep in different species^49^,^50^, whereas regulation of 5-HT2A receptors modulates slow-wave sleep (SWS)^51^,^52^. In addition, 5-HT innervation has been reported to be predominantly non-junctional; that is, localization of 5-HT receptors has been observed where receptor-expressing neurons lack direct synaptic contact in brain areas such as the neostriatum, cerebral cortex, and hippocampus^53^,^54^. This suggests that 5-HT can behave as a neuromodulator and use volume transmission as a mode of communication. As a consequence, the neuromodulator effect of 5-HT on glutamatergic and GABAergic neurons has been reported in different brain regions^24^. The present study also suggests that 5-HT neurons have a similar mode of neurotransmission in the hypothalamus and, thus, may regulate multiple types of neurons residing in this brain region. This phenomenon of 5-HT neuron activity may further complicate its regulatory actions on orexin neurons in the LHA. Therefore, additional studies are needed to determine the 5-HT receptor subtypes expressed in GABAergic neurons in the LHA, which directly innervate orexin neurons.

Strong expression of microbial opsins is required to activate nerve terminals that are devoid of cell bodies using optogenetics. However, *in vitro* and *in vivo* studies have already been conducted that demonstrate feasibility using ChR2 to control neurotransmitter release from nerve terminals^55^,^56^. As such, we showed that 5-HT neurons inhibit orexin function by facilitating IPSCs in addition to direct hyperpolarization. Consequently, activating 5-HT nerve terminals in the LHA decrease the firing probability of orexin neurons. However, optogenetic activation fails to completely silence orexin neurons, and causes only a 30% decrease in firing frequency. Our previous study showed that orexin neurons are activated by orexin and acetylcholine^29^,^31^. These interact as a positive feedback loop since orexin neurons innervate cholinergic neurons in the basal forebrain and receive cholinergic input. Moreover, orexin neurons directly innervate and activate other orexin neurons in the LHA via OX2Rs^29^,^31^. This neurocircuit functions to reinforce its own activity, which might play a role in maintenance of vigilance states^29^. Thus, negative regulation of this positive feedback circuit is important for maintaining neuronal firing in a moderate range, and the sustained inhibition in firing frequency of orexin neurons via 5-HT neurons also supports this hypothesis. Increased inhibitory inputs to orexin neurons by 5-HT were consistent during pharmacological application of 5-HT. Thus, we conclude from this study that 5-HT is the major neurotransmitter secreted by 5-HT neurons in the raphe nuclei that innervate orexin neurons in the LHA. Finally, our success in developing a new transgenic mouse could be very useful for further application to both *in vitro* and *in vivo* studies where other physiological roles related to orexin and 5-HT neurons need to be elucidated.

## Materials and Methods

### Animal usage

All experimental protocols that involved animals in this project were approved by the Institutional Animal Care and Use Committees, Research Institute of Environmental Medicine, Nagoya University, Japan. All experiments in this study involving the use of mice were performed in accordance with approved guidelines of the Institutional Animal Care and Use Committees, Research Institute of Environmental Medicine, Nagoya University, Japan. All efforts were made to reduce the number of animals used and also to minimize the suffering and pain of animals. Animals were maintained on a 12-hour light-dark cycle (lights were turned on at 8:00 am), with free access to food and water.

### Generation of new *Tg* mice

To control the activity of 5-HT neurons, a variant of channelrhodopsin2 (ChR2 (E123T/T159C) fused with enhanced yellow fluorescent protein (EYFP)) was exclusively expressed in 5-HT neurons under the control of the tryptophan hydroxylase 2 (tph2) promoter^57^,^58^. In this approach, the tph2 promoter drives tetracycline-controlled transactivator (tTA) expression in 5-HT neurons in the central nervous system. The tetracycline responsive element (TRE), which includes a tetracycline operator (TetO) sequence and a minimal promoter, drives the expression of a downstream gene in the presence of the tTA in the absence of doxycycline (Tet-off system)^28^. To identify orexin neurons, enhanced green fluorescent protein (EGFP) was expressed in orexin neurons under control of the human prepro-orexin promoter^31^. To achieve this, triplegenic mice, *orexin-EGFP; Tph2-tTA; TetO ChR2* were generated. At first, bigenic mice, *Tph2-tTA*; *TetO ChR2 mice,* were generated by breeding *Tph2-tTA* mice^58^ with *TetO* ChR2 mice^59^. A sufficient amount of ChR2 expression is important to establish control over neural systems using optogenetics. To achieve this, the expression of ChR2 in these mice was enhanced by knockin-mediated enhanced gene expression by an improved tetracycline-controlled gene induction (KENGE-tet) strategy^57^. In this strategy, the insertion site of TetO ChR2-EYFP was just after the polyA signal of the allele of the housekeeping gene, β-actin. Therefore, this approach allowed us both the specific and sufficient expression of ChR2 in targeted neurons. Male and female heterozygote bigenic mice, *Tph2-tTA*; *TetO ChR2,* were mated to generate homozygote bigenic mice (F1 generation). The F1 generation of the bigenic mice was screened by quantitative real time PCR (qPCR) to get double homozygote *Tph2-tTA (Tg/Tg*)*; TetO ChR2 (KI/KI*) mice. Previously, we also generated *orexin-EGFP (Tg/Tg*) mice^30^. To obtain the triplegenic mice, we crossed the homozygous bigenic mice with homozygous *orexin-EGFP* mice (Fig. 1a). All the littermates of triplegenic pups were genotyped by PCR to confirm the expression of all three transgenes using the following primer pairs: *Tph2-tTA (tTA* forward 5′-CCTGTACTGGCACGTGAAGA-3′; *tTA* reverse 5′-CCAGGGTCTCGTACTGCTTC-3′), *TetO ChR2 (TetO* forward 5′-AGCAGAGCTCGTTTAGTGAACCGT-3′; *TetO* reverse 5′-AAGGCAGGATGATGACCAGGATGT-3′) and *orexin-EGFP (orexin* forward 5′-AAGTCGACGGTGTCTGGCGCTCAGGGTG-3′; *orexin* reverse 5′-GCAGCGGCCATTCCTTGG-3′).

### Immunohistochemistry

Triplegenic mice of both sex at 3–5 months of age were deeply anesthetized with 10% somnopentyl (Kyoritsu Seiyaku Corporation, Tokyo, Japan) diluted in saline (1.0 ml/kg body weight) and were perfused transcardially with 20 ml of ice-cold saline. This perfusion was immediately followed by another using 20 ml of ice-cold formalin (10%) solution (Wako Pure Chemical Industries, Ltd., Osaka, Japan). Next, the brain was quickly removed and post-fixed by immersing in 10% formalin solution at 4 °C overnight. Subsequently, the brain was immersed in 30% sucrose in PBS at 4 °C for at least 2 days. Coronal brain slices of 40 μm thickness were generated using a cryostat (Leica CM3050 S; Leica Microsystems, Wetzlar, Germany) and slices were preserved in a phosphate-buffered saline (PBS) containing 0.02% of NaN_3_ at 4 °C until stained. For staining, coronal brain sections were immersed in blocking buffer (1% BSA and 0.25% Triton-X in PBS), then incubated with primary antibodies at 4 °C overnight. The sections were then washed by blocking buffer three times, and then incubated with secondary antibodies for 1 hr at room temperature (RT). After washing by the same blocking solutions three times, brain sections were mounted and examined with a fluorescence microscope (BZ-9000, Keyence, Osaka, Japan or IX71, Olympus, Tokyo, Japan) and/or confocal microscope (LSM 710, Zeiss, Jena, Germany).

### Antibodies and stains

Primary and secondary antibodies were diluted in the blocking buffer as follows: anti-GFP mouse antibody (Wako, Japan) at 1:1000, anti-Tph sheep antibody (Millipore, Billerica, MA) at 1:1000, anti-orexin-A goat antibody (Santa Cruz, Dallas, TX) at 1:1000, anti-HTT guinea pig antibody (Frontier Institute Co. Ltd., Hokkaido, Japan) at 1:500, CF 488-conjugated anti-mouse antibody (Biotium Inc., Hayward, CA) at 1:1000, CF 594-conjugated anti-sheep or anti-guinea pig antibody (Biotium) at 1:1000, and CF 647-conjugated anti-goat antibody (Biotium) at 1:1000.

### Acute brain slice preparation for electrophysiology

Both male and female triplegenic mice, 3–8 months old, were used for electrophysiological recordings. Mice were deeply anesthetized using isoflurane (Wako, Japan) and were decapitated. Brains were then quickly removed and chilled in an ice-cold cutting solution (in mM: 110 K-gluconate, 15 KCl, 0.05 EGTA, 5 HEPES, 26.2 NaHCO_3_, 25 Glucose, 3.3 MgCl_2_ and 0.0015 (±)-3-(2-Carboxypiperazin-4-yl)propyl-1-phosphonic acid) gassed with 95% O_2_ and 5% CO_2_. Coronal brains slices of 300 μm thickness that contained either raphe nucleus or hypothalamus were generated using a vibratome (VTA-1200S; Leica, Wetzlar, Germany). Brain slices were then temporarily preserved in an incubation chamber containing bath solution (in mM: 124 NaCl, 3 KCl, 2 MgCl_2_, 2 CaCl_2_, 1.23 NaH_2_PO_4_, 26 NaHCO_3_ and 25 Glucose) gassed with 95% O_2_ and 5% CO_2_ in a 35 °C water bath. Slices were then incubated at RT (24–28 °C) with the same incubation chamber for 30–60 minutes. Cutting and bath solutions were modified from Pressler and Regehr (2013)^60^.

### Electrophysiological recordings

Acute brain slices were transferred into a recording chamber (RC-27L; Warner Instruments, Hamden, CT) on a fluorescence microscope (BX51WI; Olympus Optical, Japan) stage and were superfused with the gassed (95% O_2_ and 5% CO_2_) bath solution at the rate of 1.0 ml/min using a peristaltic pump (Dynamax, Rainin, USA). An infrared camera (C2741-79; Hamamatsu Photonics, Hamamatsu, Japan) was installed in the fluorescence microscope along with an electron-multiplying charge-couple device camera (Evolve 512 delta; Photometrics, Tucson, AZ), and both images were separately displayed on monitors. A micropipette puller (P-1000; Sutter Instrument, Novato, CA) was used to prepare patch pipettes (GD150-10, Harvard Apparatus, Cambridge, MA) of 5–8 MΩ resistance. Patch pipettes were filled with a KCl-based internal solution (in mM: 145 KCl, 1 MgCl_2_, 10 HEPES, 1.1 EGTA, 2 Mg-ATP, 0.5 Na_2_-GTP; pH 7.3 with KOH) with the osmolarity checked to be in between 280–290 mOsm. Orexin neurons were located by green fluorescence from EGFP and serotonergic neurons were found by green fluorescence from EYFP. Although both neurons displayed green fluorescence, orexin neurons and 5-HT neurons are easily distinguishable from each other since the distribution of cell bodies of orexin neurons and 5-HT neurons is largely restricted to the hypothalamus and raphe nucleus, respectively. Positive pressure was introduced in the patch pipette before it was advanced toward the cell. For voltage or current clamp recordings, a giga-seal (resistance >1 GΩ) was made between the patch pipette and the cell membrane by releasing the positive pressure upon contacting the cell. The patch membrane was then ruptured by gentle suction to form a whole-cell configuration. A loose cell-attached configuration was made by forming a loose-seal rather than a giga-seal with the cell membrane. Electrophysiological properties of cells were monitored by an Axopatch 200B amplifier (Axon Instrument, Molecular Devices, Sunnyvale, CA). Output signals were low-pass filtered at a 5 kHz and digitized at a 10 kHz sampling rate. Patch clamp data were recorded through an analogue-to-digital (AD) converter (Digidata 1322A; Axon Instruments) using pClamp 10.2 software (Molecular Devices). Blue light of 475 ± 17.5 nm wavelength was generated by a light source that uses a light-emitting diode (Spectra light engine; Lumencor, Beaverton, OR) and was guided to the microscope with a liquid light fibre of 1 cm diameter. Brain slices were illuminated through the objective lens of a fluorescence microscope. Blue light intensity (in %) of 1, 2, 5, 10, 20, 50, and 100 indicates light power (in mW) of 0.01, 0.3, 1.3, 2.5, 4.7, 9.7, and 15.4, respectively. Evoked EPSCs were recorded with picrotoxin (400 μM) and IPSCs were recorded with AP-5 (50 μM) and CNQX (20 μM) in bath solutions. Both EPSCs and IPSCs were recorded in the presence of KCl-based pipette solutions that included 1 mM of QX-314. 5-HT (100 μM) was applied locally using a six-channel perfusion valve controller (VC-6, Warner Instruments) and were washed out by bath solutions.

### Quantification

Quantification of cells was performed on ImageJ (US National Institute of Health). The cell counter plugin was used to aid counting. First, Tph-positive neurons were counted and then were evaluated for co-expression of the GFP^+^ marker. To determine the percentage of 5-HT neurons expressing ChR2, the counted number of GFP^+^ cells was divided by the total number of serotonergic neurons on a given slice and were multiplied by 100. Cells positive for ChR2 but Tph-negative were also checked to determine any ectopic expression.

### Data analysis and presentations

Imaging data were analysed and processed with ImageJ (US National Institute of Health), BZ-X Analyzer (Keyence BZ-X710 microscope), or ZEN (Carl Zeiss Microscopy). Analysis of electrophysiological data was done by Minianalysis software (Synaptosoft Inc., Decatur, GA). After analysis, electrophysiological data were saved as American Standard Code for Information Interchange or ASCII files and further data calculations were done in Microsoft Excel. Graphs were generated in OriginPro8.1J (OriginLab, Northampton, MA) using data from Excel. Statistical analysis was also done in OriginPro8.1J. Finally, some graphs were generated on Canvas 15 (ACD systems, Seattle, WA). To measure the cumulative probability of synaptic events, the Kolmogorov-Smirnov test was used. All statistical tests including exact *p* values were described when used. No statistical analyses were used to predetermine sample sizes. All data were presented as the mean ± standard error of the mean (s.e.m.). For all statistical tests *p < 0.05, **p < 0.01, ***p < 0.001 were considered significant and *p* > 0.05 was considered not significant (*ns*).

## Additional Information

**How to cite this article**: Chowdhury, S. and Yamanaka, A. Optogenetic activation of serotonergic terminals facilitates GABAergic inhibitory input to orexin/hypocretin neurons. *Sci. Rep.* **6**, 36039; doi: 10.1038/srep36039 (2016).

**Publisher’s note:** Springer Nature remains neutral with regard to jurisdictional claims in published maps and institutional affiliations.

## Supplementary Material

Supplementary Information
