# Supplementary material for: Optogenetic activation of serotonergic terminals facilitates GABAergic inhibitory input to orexin/hypocretin neurons
Source: Sci Rep. 2016 Nov 8;6:36039. doi: 10.1038/srep36039 (PMC5099903; doi:10.1038/srep36039)
Supplement: Supplementary Information [file srep36039-s1.pdf]

Optogenetic activation of serotonergic terminals facilitates GABAergic inhibitory input to orexin/hypocretin neurons

Srikanta Chowdhury[1] and Akihiro Yamanaka[1*]

[1] Department of Neuroscience II, Research Institute of Environmental Medicine, Nagoya University, Nagoya 464-8601, Japan

[*]Corresponding author:

Akihiro Yamanaka Ph.D.

Department of Neuroscience II, Research Institute of Environmental Medicine

Nagoya University, Nagoya 464-8601, Japan

Tel: +81-52-789-3861

Fax: +81-52-789-3889

E-mail: yamank@riem.nagoya-u.ac.jp

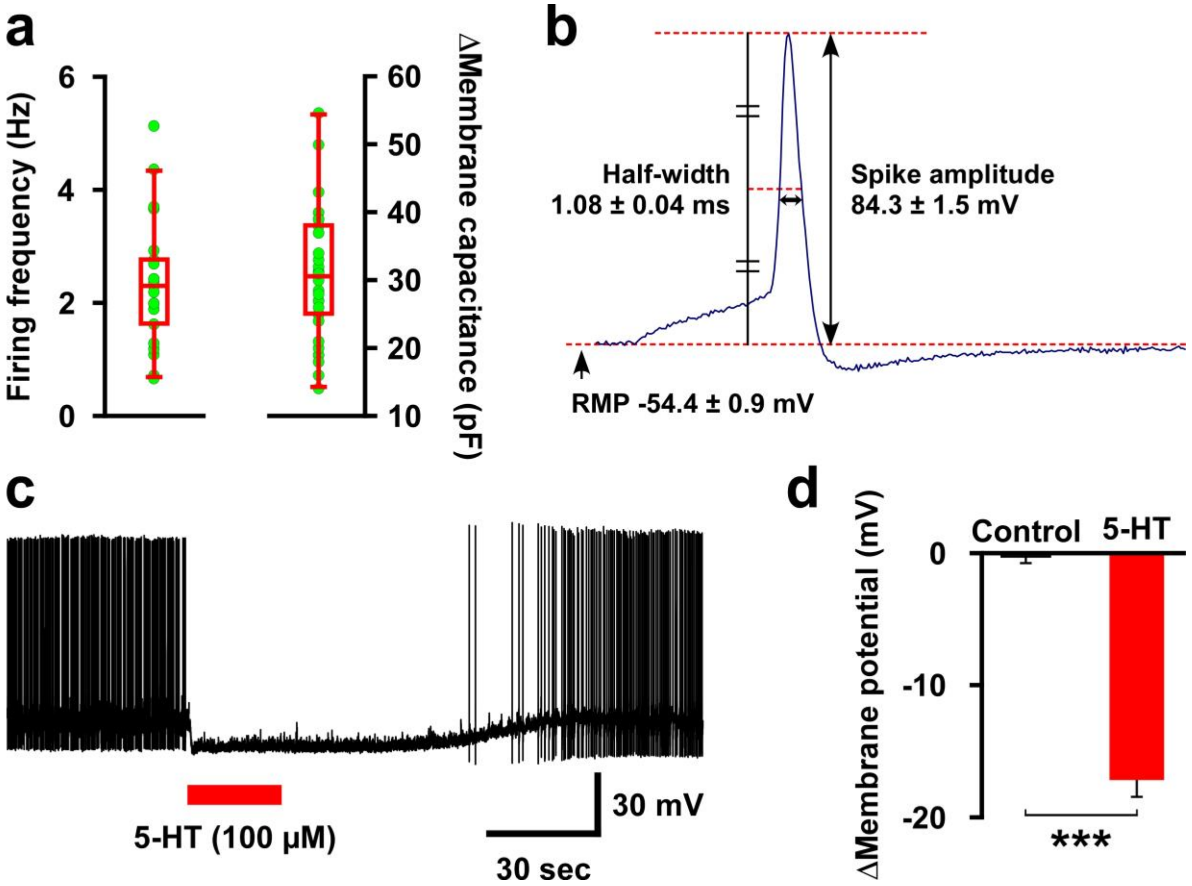

Supplementary Figure 1. Electrophysiological properties of orexin neurons in triplegenic mice. **(a)** Left: Spontaneous firing frequency recorded by loose cell-attached recording from orexin neurons (n = 21). Right: Averaged membrane capacitance of orexin neurons recorded from whole-cell experiments (n = 30). Green circles indicate raw data. The mid-horizontal line indicates the median, box gives the 25 and 75%, and whisker indicates 10 and 90% ranges of data. **(b)** Shape of orexin neuron action potential in the triplegenic mice. Average resting membrane potential, action potential amplitude, and width at half-maximal amplitude are indicated (n = 15-23). **(c, d)** Local application of 100 μM 5-HT hyperpolarizes orexin neurons. (**c**) Typical trace showing hyperpolarizing effect of 5-

HT on orexin neurons. (**d**) Bar diagram summarizing data in **c** (*n* = 19). Data are provided as mean ± s.e.m. ($p = 3.2 \times 10^{-10}$ by two-tailed Student's *t* test).

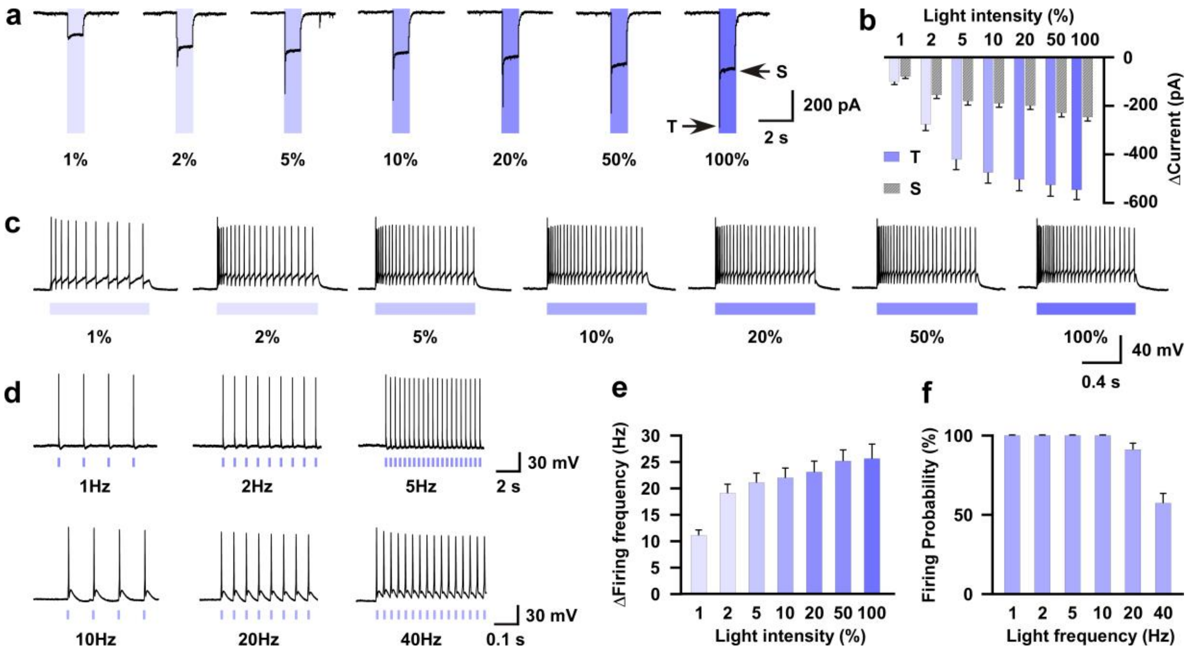

Supplementary Figure 2. *In vitro* optogenetic activation of 5-HT neurons in the raphe nucleus. **(a, b)** Photocurrent data from whole-cell voltage clamp recordings at -60 mV_{hold}. T = Transient current and S = Sustained current. **(a)** In the presence of QX-314 in the intracellular solution, both transient and sustained currents were increased in a light-intensity dependent manner upon illuminating blue light stimulation (475 ± 17.5 nm). (**b)** Summary of data in **a** (*n* = 11). **(c-f)** Whole-cell current clamp recordings from 5-HT neurons. Blue light evoked action potentials during (**c)** continuous application (1 s) of blue light with different intensities and (**d)** blue light pulses (1 ms width and 10% intensity) with different frequencies. **(e)** Summary of data in **c** shows firing frequency increased in a light-intensity dependent manner (*n* = 11). **(f)** Summary of data in **d** shows percent of faithful firing (*n* = 10). All data are shown as mean ± s.e.m. Transient currents were measured immediately after light onset and sustained currents were measured just before cessation of light. 5-HT neurons were chosen randomly from both DR and MnR.

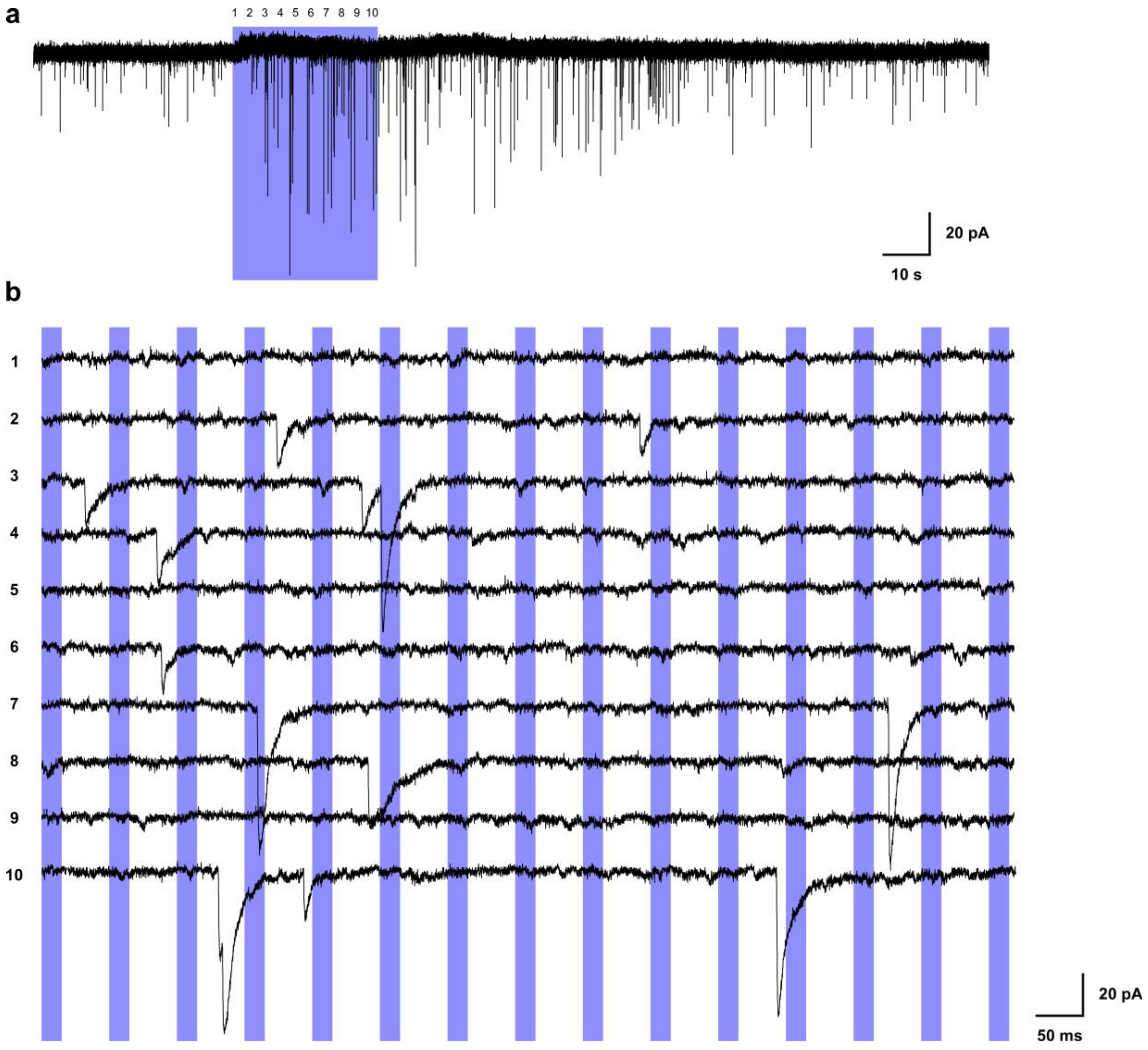

Supplementary Figure 3. IPSCs in orexin neurons evoked by optogenetic activation of 5-HT nerve terminals are not induced in a precise time-locked manner. (a) Trace showing light-evoked IPSCs in orexin neurons at -60 mV_{hold} that were generated by 15 Hz blue light pulses of 20 milliseconds (ms) width and 50% light intensity. (b) Ten enlarged current traces from the traces shown in (a) taken at a defined interval (1 sec trace from every 3 sec) shows that the frequency and amplitude of IPSCs were increased after blue light pulse. However, IPSCs were not precisely time-locked to the blue light pulses.

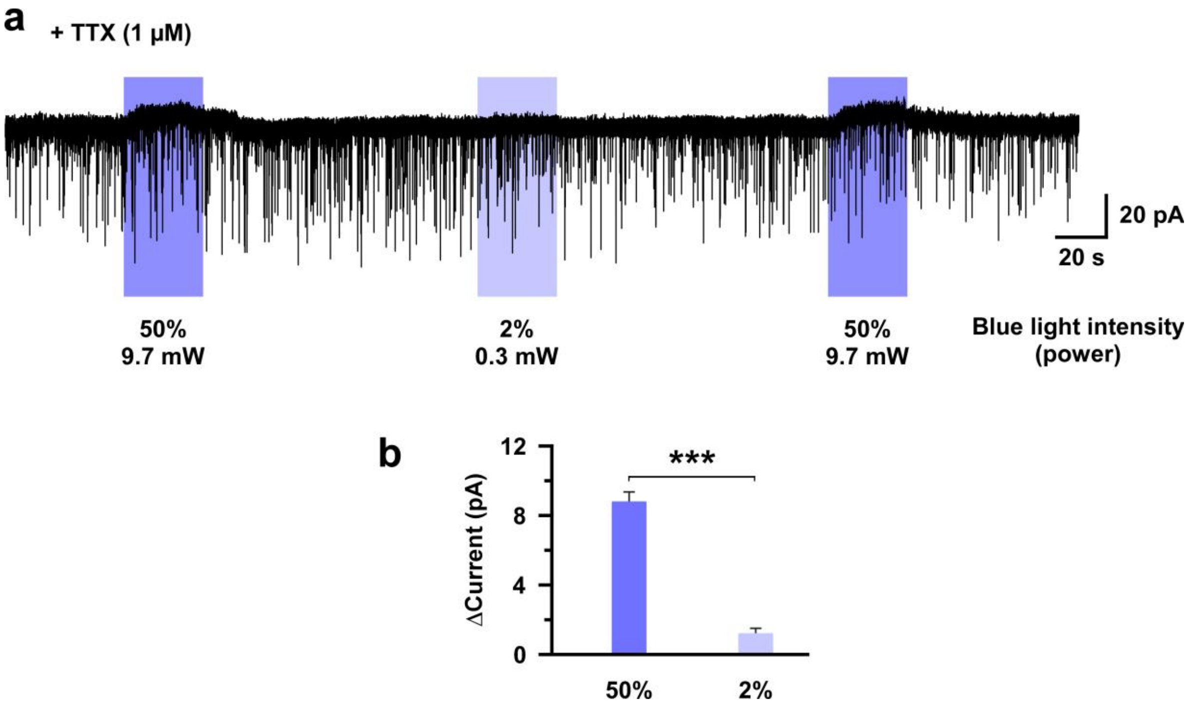

Supplementary Figure 4. The post synaptic effect of activating 5-HT nerve terminals depends on the blue light intensity. (a) Traces showing outward currents in orexin neurons generated by blue light of different intensities. (b) Bar graph summarizing the data from experiments shown in (a) (n = 8). Data are provided as the mean ± s.e.m. ($p = 1.3 \times 10^{-7}$ by two-tailed Student's t test).
